# The comparison between total hip arthroplasty and hemiarthroplasty in patients with femoral neck fractures: a systematic review and meta-analysis based on 25 randomized controlled trials

**DOI:** 10.1186/s13018-020-02122-6

**Published:** 2020-12-10

**Authors:** Xiumei Tang, Duan Wang, Ying Liu, Jiali Chen, Zongke Zhou, Peifang Li, Ning Ning

**Affiliations:** 1 West China School of Nursing, Sichuan University/Department of Orthopedics, West China Hospital, Sichuan University, Chengdu, 610041 People’s Republic of China; 2 Department of Orthopedics, Orthopedic Research Institute, West China Hospital, Sichuan University, Chengdu, 610041 People’s Republic of China

**Keywords:** Total hip arthroplasty, Hemiarthroplasty, Femoral neck fractures, Randomized controlled trials

## Abstract

**Background:**

We performed an updated systematic review and meta-analysis which enrolled 25 prospective randomized controlled trials (RCTs) to compare the outcomes between total hip arthroplasty (THA) and hemiarthroplasty (HA) in patients with femoral neck fractures (FNFs).

**Methods:**

We searched English databases which included PubMed, Embase (vis OvidSP), The Cochrane Library, and Web of Science, and Chinese databases Chinese National Knowledge Infrastructure (CNKI), VIP, Wang Fang, and China Biology Medicine Disc (CBM) in July 2020. The quality of each study was assessed according to the Cochrane Collaboration’s Risk of Bias. Risk ratios (RRs) and weighted mean differences (WMDs) with 95% confidence intervals (95% CIs) were pooled with random-effects models. Data regarding baseline characteristics, hospital and surgery outcomes, clinical outcomes, patients’ quality of life, common complications, prothesis-related complications, mortality, and costs were reported.

**Results:**

A total of 25 RCTs involving 3223 patients (1568 THA and 1655 HA) were included. THA had longer hospital length (WMD = 0.721, *P* < 0.0001) and surgery time (WMD = 20.044, *P* < 0.0001), and more blood loss compared with HA (WMD = 69.109, *P* < 0.0001). THA showed better ratings in the Harris Hip Score during follow-up periods between 1 and 5 years while no differences within 6 months and after 9 years. THA was associated with higher quality-of-life EuroQol-5 Dimension (EQ-5D) scores after 2 years of surgery but no difference within 1 year. There was no difference in common complications. THA had significant higher rate of dislocation (WMD = 1.897, *P* = 0.002) and lower acetabular erosion (WMD = 0.030, *P* = 0.001). For mortality, there was no difference during all the follow-up periods except for slightly higher 2-year mortality after surgery.

**Conclusion:**

This meta-analysis demonstrates that THA has better medium-term functional results and quality of life and lower acetabular erosion rate, while HA shows better in reducing hospital stay, surgery time, and blood loss and also has lower dislocation rate.

## Background

Femoral neck fractures (FNFs) will bring baneful influences to patients due to its high morbidity, disability rate, economic burden, and mortality, and the rate is rapidly growing because of the increasing number of the elderly [23]. Arthroplasty is commonly recommended for displaced femoral neck fractures (67% of all types FNFs) in the elderly (age > 65 years) and can be categorized as total hip arthroplasty (THA) and hemiarthroplasty (HA) [34]. Whether THA or HA is more applicable in FNF remains controversial [21]. Both pros and cons of the treatments were widely reported in previous studies and synthesized reviews but did not reach a common conclusion [6, 11, 13, 15, 17, 24, 26, 33, 49–52]. The ongoing discussion requires highly reliable answers. However, previous meta-analysis and reviews have several limitations. First, they did not fully mention the details of surgical approach, prosthetic choice, surgeon experience, and the type of both femoral and acetabular fixation, all of which we consider may cause chaos in conclusion. Second, serious inclusion criteria in some studies may lead to limited data to analyze. Third, subgroup analysis was limited, and long-term results were not considered. The latest meta-analysis included trials reported between 2006 and 2017 and may be outdated [33]. Randomized controlled trials (RCTs) with high quality have been published recently and not been included, and we carefully selected Chinese articles reported with enough follow-up duration and reported outcomes in our analysis [3, 8, 18, 25, 27, 31, 38, 44].

We conducted an updated meta-analysis only including RCTs to provide the most reliable evidence.

## Methods

The review followed Preferred Reporting Items for Systematic Reviews and Meta-Analyses (PRISMA) guidelines (www.prisma-statement.org).

### Searches and selection criteria

We searched English databases which included PubMed, Medline, Embase, The Cochrane Library, and Web of Science and Chinese databases CNKI, VIP, WAN FANG, and CBM (all inception to July 2020) without language or date restriction as well as retrieving articles identified in other reviews by manual search. And the search strategy is provided in Supplementary files. Inclusion criteria were RCTs comparing THA with HA for FNFs and at least reporting one of the predetermined outcomes. To make our conclusion generalizable, we set no restrictions for follow-up time, patients’ age, study size, or pre-surgery status.

### Outcome measures

We included the following outcomes:

- Hospital and surgery outcomes: hospital stays, surgery duration, blood loss;
- Clinical outcomes: Harris Hip Scores (HHS) within 6 months and up to 13 years;
- Patients’ quality of life: EQ-5D scores within 6 months and at 1 to 2 years;
- Common complications: pulmonary embolism, deep vein thrombosis, pneumonia, urinary tract infection, pressure ulcer, wound disease, surgical-site infection, and cardiovascular disease.
- Prothesis-related complications: revision, fracture, dislocation, loosening or subsidence, heterotopic ossification, and acetabular erosion;
- Mortality: mortality in hospital, within 6 months, at 1 to 2 years and up to 13 years;
- Cost;

### Data extraction and study quality assessment

Two reviewers (T-XM, WD) independently screened the titles and abstracts for eligibility. We develop a data extraction form and collected data from including articles after full-text reading and cross-checking procedures. Any discrepancies were evaluated by a third reviewer(C-JL). For study quality assessment, the Cochrane Collaboration’s Risk of Bias was used. For missing data like standard deviation, we calculated them with formulas according to the Cochrane handbook for systematic reviews of interventions or articles’ figure data.

### Statistical methods

For statistical analysis, the review used forest plots to present the synthesized results. For continuous and binary variables, the weighted mean differences (WMD) and risk ratios (RR) were reported respectively with 95% confident interval (CI). Survivorship was analyzed through the Kaplan–Meier survivor curve. Heterogeneity was assessed by both *Q*^2^ and *I*^2^ tests, and *P* value < 0.1 or *I*^2^ > 50% indicates statistical heterogeneity. Galbraith tests and sensitivity analysis were used to identify the possible heterogeneity origins. If necessary, subgroups will be used to dismiss heterogeneity. The random effects model was conducted in any condition. We used sensitivity analysis by sequential omission of individual studies to validate the credibility of pooled data. For publication bias, the symmetry of funnel plots was visually evaluated, and Egger’s tests were also applied. For statistical analyses, the Review Manager (Version 5.0.2) and STATA (Version 13.0) software programs. All *P* values were two-sided.

## Results

### Search results

**Fig. 1:**
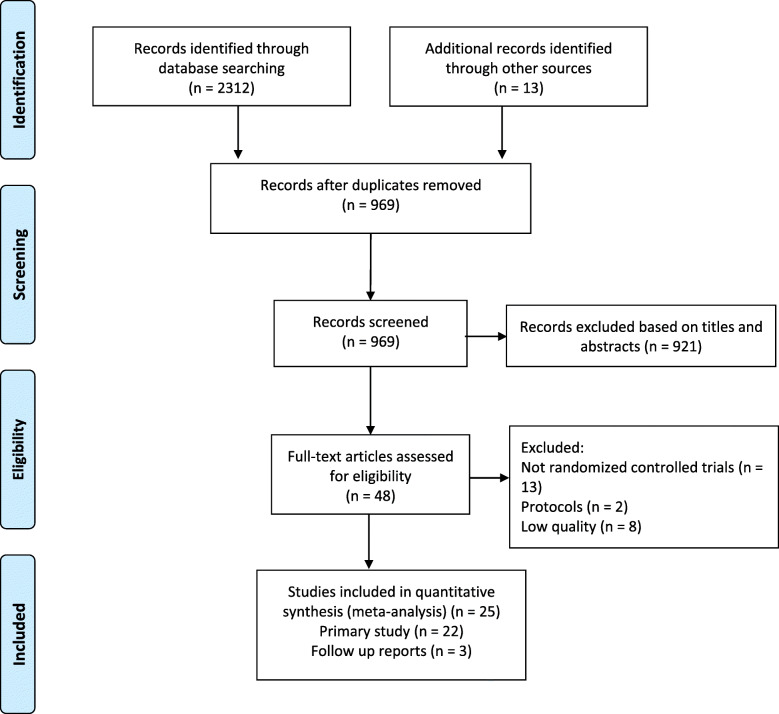
PRISMA flowchart of the selection process

Our review yielded 2325 reports and excluded 1356 after duplicates. Of these literatures, 48 were included after selecting the title and abstracts. After full text screening, 23 were excluded, and the details were described in the flow chart (Fig. 1). For clinical outcomes, we included 25 reports based on 19 trials and extracted non-repeating data at different follow-up stages ([1–4, 7, 8, 10, 12, 16, 18, 22, 25]; H. H [27].; W [29].; William [30, 31, 36–38, 40, 42–44, 46, 47]).

### Methodological quality assessment

**Fig. 2:**
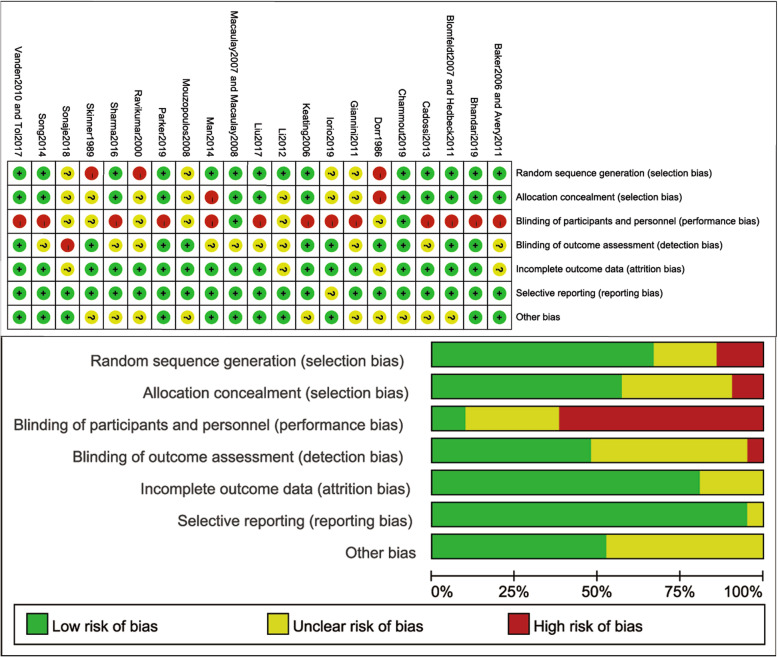
Risk of bias of included studies according to the Cochrane Collaboration’s Risk of Bias

In this study, selection, attrition, and reporting bias can be considered low risk. Detection bias was moderate risk as well as performance bias. Therefore, the methodological assessment of this work can be judged as very good quality. Two reviewers independently assessed the risk of bias of included studies according to the Cochrane Collaboration’s Risk of Bias, and the results are shown in Fig. 2.

### Risk of publication bias

**Fig. 3:**
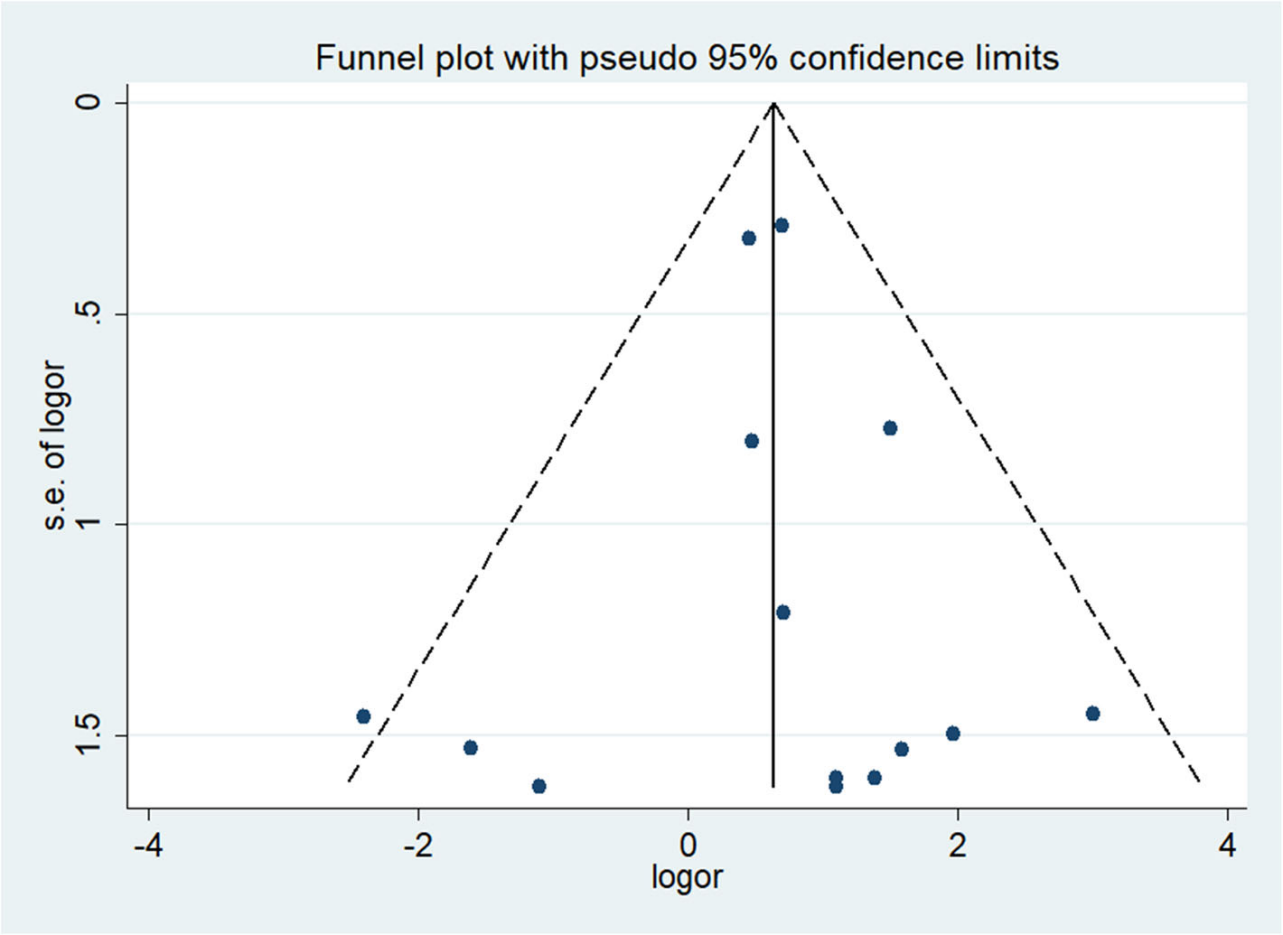
Funnel plot based on dislocation rate

Funnel plots of the outcome enrolled the most studies (dislocation) to detect publication bias. The symmetrical distribution and Egger’s test (*P* = 0.708) show low publication bias (Fig. 3).

### Study characteristics

We finally included 25 RCTs involving 3223 patients (THA 1568, HA 1655). Five of them ([1, 16]; W [29, 40].) were follow-up reports of previous trials. Table 1 summarizes the trials’ details.

**Table 1:** Characteristics of the included studies

Study	Country	Period	Surgical approach	Surgeon	Patients number		Age		Woman		ASA		Median time to surgery, h		Mobility	Mental status	THA	HA	Follow-up
					THA	HA	THA mean (SD)	HA mean (SD)	THA *n* (%)	HA *n* (%)	THA	HA	THA	HA					
Baker et al. [2] and Avery et al. [1]	England	N/A	Transgluteal lateral	Similar training levels	40	41	74.2 (5.8)	75.8 (5)	32 (80%)	32 (78%)	2 (0.5)	2 (0.5)	42	46.8	Walk > 0.8 km; live independently	MMSE9.83/9.98	**Zimmer; femoral:** cemented; **head** 28 mm cobalt chrome; **acetabular:** Polyethylene cemented	Zimmer; cemented; unipolar	3 years for Baker et al. [2]; 9 years for Avery et al. [1]
Bhandari et al. [3]	Multiple centers	2009–2017	Not standardize	523 surgeons with expertise in THA/HA	718	723	79.1 (8.3)	78.6 (8.6)	510 (71%)	499 (69.1%)	22/280/305/50	20/275/326/51	54.9(79)	52.5(80.3)	1072 (walk dependently)/369 (with assistance)	No dementia	Mixed	Mixed	2 years
Blomfeldt et al. [4] and Hedbeck et al. [16]	Sweden	N/A	Anterolateral	9 surgeons experienced in THA/HA	60	60	80.5 (4.9)	80.7 (4.8)	47 (78%)	54 (90%)	N/A	N/A	N/A	N/A	111 no aids or one aid (92.5%)	SPMSQ > 9	**Johnson; femoral:** cemented; **head** 28 mm exter modular stem; **acetabular:** OGEE, DePuy	Stryker cemented; bipolar	12 months for Blomfeldt et al. [4]; 48 months for Hedbeck et al. [16]
Cadossi et al. [6]	Italy	2008–2010	Straight lateral	2 experienced surgeons (SG, CF)	42	41	82.3 (6.3)	84.2 (6.3)	34 (81%)	28 (68%)	2/15/16/9	1/10/22/8	2.9 (1.75)	3.6 (1.5)	Independently walk	No senile dementia	**Unknown** **Femoral:** uncemented; **head:** large metal; **acetabular:** 2.7-mm thick hydrophilic polycarbonate urethane (PCU)	Mixed; bipolar	30.1 months
Chammout et al. [8]	Sweden	2009–2016	Direct lateral	Consultant surgeon or registrar with assistance of consultant	56	62	85 (4)	86 (4)	45 (75%)	45 (75%)	ASA1/2:30; ASA3,4:30	ASA1/2:20; ASA3,4:40	< 36 h	< 36 h	No aids or one aid: THA30 (50%)/HA29 (48%)	SPMSQ:8-10	**Schering-Plough** **Femoral:** cemented; (vacuum-mixed low-viscosity cement with gentamicin);**head** 32-mm cobalt-chromium; **acetabular:** cemented highly cross-linked polyethylene	Cemented; unipolar	2 years
Dorr et al. [10]	America	1980–1982	Posterior	N/A	39	50	69 (9)	69 (12)	23 (60%)	35 (70%)	N/A	N/A	N/A	N/A	Ambulate	Mental status1/2: 70/19	**Unknown** **Femoral:** cemented; **head:** 28-mm; **acetabular:** unknown	Mixed; bipolar	2 years
Giannini et al. [12]	Italy	N/A	N/A	N/A	30	30	80.7 (6)	82.2 (6)	N/A	N/A	N/A	N/A	N/A	N/A	N/A	N/A	**Unknown** **Femoral:** uncemented; **head:** large metal; **acetabular:** pliable, 3-mm-thick polycarbonate-urethane (PCU)	Mixed; bipolar	1 year
Iorio et al. [18]	Italy	2015–2017	Direct lateral	N/A	30	30	82 (4)	83 (3)	18 (60%)	17 (56.7%)	0/3/23/4	0/4/21/5	59 (13)	51 (15)	Walk unaided	MMSE < 18	**Groupe Lépine** **Femoral:** Uncemented; **head:** dual mobility cup Quattro; **acetabular: N/A**	Uncemented; bipolar	2 years
Keating et al. [22]	England	1996–2000	Decided by surgeon	Senior surgeon	69	111	75.2 (6)	75.4 (7)	52 (75%)	92 (83%)	N/A	N/A	< 48 h	< 48 h	Independent	MMSE > 6	**Unknown** **Femoral:** cemented; **head:** N/A; **acetabular:** N/A	Cemented; bipolar	2 years
Li et al. [25]	China	2010–2012	Anterolateral	N/A	40	40	76.5 (6.5)	75.8 (6.2)	17 (42.5%)	19 (47.5%)	N/A	N/A	N/A	N/A	N/A	Eliminate cognitive impairment	**Unknown** **Femoral:** mixed; **head:** N/A; **acetabular:** N/A	Mixed; N/A	2 years
Liu et al. [27]	China	2010–2012	Posterior	N/A	54	54	74.19(6.4)	75.31 (6.2)	24 (44.44%)	26 (48.1%)	N/A	N/A	N/A	N/A	N/A	Excluded dementia patients	N/A	N/A	1 year
Macaulay et al. [30] and Macaulay et al. [29]	America	N/A	Posterolateral and direct lateral (Modified Hardinge)	14 surgeons, 5 reconstruction specialists	17	23	82 (7)	77 (9)	7 (41%)	14 (61%)	N/A	N/A	N/A	N/A	Independently walk	Excluded MMSE < 23	Mixed	Mixed	1 year for Macaulay et al. [30]; 2 years for Macaulay et al. [29]
Man et al. [31]	China	2010–2012	Moore approach	N/A	37	37	N/A	N/A	28 (37.84%)	28 (37.84%)	N/A	N/A	N/A	N/A	N/A	No cognitive impairment	Cemented; N/A	Mixed; bipolar	3 years
Mouzopoulos et al. [36]	Greece	1999–2002	N/A	N/A	37	34	73.07(4.9)	74.24 (3.77)	28 (75.68%)	24 (70.59%)	2.03 (1.97)	2.21 (1.9)	45.2 (7.3)	45.8 (2.4)	Independently walk 37/34	SPMSQ 7.9 (2.6)/7.5 (3.1)	N/A	N/A	4 years
Parker et al. [28]	England	2012–2018	Anterolateral	See in a.	52	53	77.1 (5.5)	77.1 (7.25)	40 (76.9%)	45 (84.9%)	2.2	2.0	N/A	N/A	Mean mobility grade 1.6/1.4 (9 grades)	MMSE 8.7/8.9			
Skinner et al. [37] and Ravikumar et al. [40, 41]	England	1984–1986	Posterolateral	See in b.	89	91	81.03	82.06	N/A	N/A	N/A	N/A	Within 24 h	Within 24 h	N/A	Included patients with dementia	**Unknown** **Femoral:** cemented (Howse II prosthesis); **head** 32 mm head; **acetabular:** semi-captive cup	Bipolar; uncemented (Austin Moore)	2 years for Skinner et al. [37]; 13 years for Ravikumar et al. [40, 41]
Sharma et al. [42]	India	2010–2014	Modified Gibson	Two senior arthroplasty surgeons	40	40	78 (3.5)	73 (1.25)	26 (65%)	29 (72.5%)	N/A	N/A	72 h	72 h	N/A	N/A	N/A	N/A	1 year
Sonaje et al. [43]	India	2011–2012	N/A	N/A	21	21	66.4 (3.5)	65.3 (3)	13 (65%)	14 (70%)	N/A	N/A	N/A	N/A	N/A	No psychiatric and neurological disorder	N/A	N/A; bipolar	2 years
Song et al. [44]	China	2003–2012	N/A	N/A	31	31	64.5 (5.8)	65.1 (5.9)	12 (38.71%)	10 (32.26%)	N/A	N/A	N/A	N/A	N/A	N/A	N/A	N/A; bipolar	1 year
Van den et al. [47] and Tol et al. [46]	Netherlands	1995–2002	Anterolateral; straight lateral; posterolateral	Experienced surgeon or residents with assistant	115	137	82.1 (6.3)	80.3 (6.2)	90 (78%)	115 (84%)	11/48/44/10	19/77/33/5	1(2.25)	1(2.5)	Walk without aids:149	N/A	**Sulzer AG/Protek AG** **Femoral:** cemented (A Weber Rotations prosthesis, Müller Geradschaft prothesis); **head** 32 mm diameter modular head**; acetabular:** semi-captive cup	Cemented; Bipolar;	5 years for Van den2010 and 12 years for Tol2017

^a^All but eight operations were directly undertaken or supervised by the lead trialist. Two hemiarthroplasties and two THR’s were undertaken by orthopedic consultants and three hemiarthroplasties and one THR by trainee or staff grade surgeons

^b^Mostly by registers, occasionally by consultants or senior house officers

^c^*SPMSQ* Short Portable Mental Status Questionnaire

#### Outcome of interests

The overall results are presented in Table 2.

**Table 2:** The results of meta-analysis

Variables	*N* (study)	N (THA)	N (HA)	Pooled data		Heterogeneity	
				WMD/RR(95%CI)	P	I^2^(%)	Ph
**Hospital and Surgery**							
Hospital length	9	418	443	2.360 (0.215, 4.506)	0.031	96.0%	< 0.0001
Hospital length (deleted Li et al. [25], Liu et al. [27])	7	324	349	0.721 (0.362, 1.080)	< 0.0001	0%	0.428
Surgery time	15	1292	1341	20.044 (14.257, 25.830)	< 0.0001	95.7%	< 0.0001
Blood loss	9	1063	1038	69.106 (39.083, 99.129)	< 0.0001	96.4%	< 0.0001
Blood loss (deleted developing countries’ studies)	4	881	856	76.027 (51.951, 100.104)	< 0.0001	17.6%	0.303
**Clinical outcomes**							
By follow-up							
HHS(< 6 months)	5	208	208	1.641 (− 0.449, 3.731)	0.124	0%	0.784
HHS (at 1 year)	6	317	333	3.593 (1.278, 5.907)	0.002	12.5%	0.335
HHS (at 2 years)	5	174	168	3.691 (0.571, 6.812)	0.020	38.8%	0.162
HHS (3 to 5 years)	5	233	251	6.027 (0.434, 11.621)	0.035	90.1%	< 0.0001
HHS (at 9 years)	2	57	71	5.848 (− 4.603, 16.299)	0.273	74.1%	0.050
Pain (HHS subscore)	3	148	148	0.065 (− 0.385, 0.515)	0.777	85.5%	< 0.0001
Pain (HHS subscore, < 6 months)	2	97	97	− 0.042 (− 0.686, 0.602)	0.897	79.5%	0.027
Pain (HHS subscore, at 1 year)	3	128	128	0.405(− 0.575, 1.385)	0.418	92.7%	0.000
Pain (HHS subscore, at 2 years)	2	57	57	− 0.020 (− 0.902, 0.862)	0.964	78.6%	0.031
EQ-5D(<6 months)	3	183	214	0.031 (− 0.031, 0.093)	0.324	2.1%	0.360
EQ-5D(at 1 year)	3	181	177	0.033 (− 0.036, 0.102)	0.351	0.0%	0.951
EQ-5D(at 2 years)	3	173	165	0.107 (0.049, 0.164)	< 0.0001	0.0%	0.525
Pain (binary)	3	827	783	0.244 (0.050, 1.183)	0.080	91.0%	< 0.0001
**Common complications**							
Pulmonary embolism	4	187	235	0.597 (0.158, 2.257)	0.447	0%	0.517
Deep vein thrombosis	8	397	439	1.004 (0.386, 2.614)	0.994	13.3%	0.326
Pneumonia	7	308	315	0.932 (0.431, 2.014)	0.858	0%	0.733
Pressure injury	4	183	185	1.233 (0.301, 5.056)	0.771	0%	0.516
Wound disease	10	1170	1227	0.857 (0.488, 1.505)	0.591	0%	0.933
Surgical site infection	5	967	974	0.963 (0.422, 2.200)	0.929	17.3%	0.305
Cardiovascular disease	6	286	335	1.474 (0.672, 3.233)	0.333	0%	0.669
**Implant-related complications**							
Revision	13	1397	1480	0.736 (0.419, 1.292)	0.286	47.2%	0.030
Revision (Deleted Ravikumar et al. [40, 41])	12	1308	1389	0.882 (0.513, 1.517)	0.651	30.3%	0.150
Fracture	7	1065	1094	1.064 (0.707, 1.600)	0.767	0%	0.853
Dislocation	16	1473	1562	1.897 (1.273, 2.827)	0.002	4%	0.407
Heterotopic Ossification	3	853	880	1.272 (0.844, 1.918)	0.251	0%	0.647
Loosening or Subsidence	2	833	860	0.640 (0.170, 2.409)	0.509	25.5%	0.247
Acetabular Erosion	2	215	238	0.030 (0.004, 0.219)	0.001	0%	0.769
**Mortality**							
By follow-up							
Mortality (in hospital)	8	414	456	1.484 (0.616, 3.579)	0.379	0%	0.434
Mortality (< 6 months)	2	69	76	0.679 (0.094, 4.892)	0.767	37.5%	0.206
Mortality (at 1 year)	7	372	394	1.011 (0.684, 1.493)	0.958	0%	0.705
Mortality (at 2 years)	8	1197	1294	1.224(1.055,1.421)	0.008	12.3%	0.334
Mortality(3 to 5 years)	2	249	270	1.138(0.869,1.490)	0.346	12.8%	0.284
Mortality(9 to 13 years)	3	435	473	1.021(0.881,1.183)	0.786	0%	0.489

##### Hospital and surgery outcomes

**Fig. 4:**
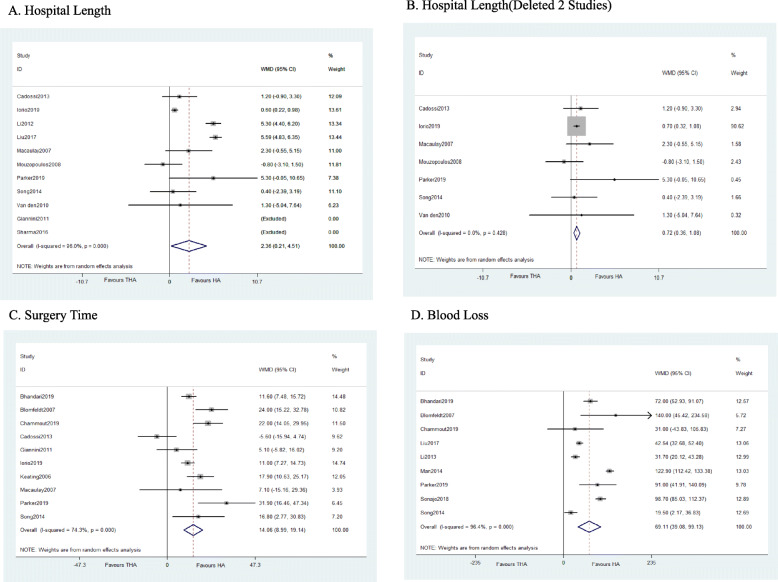
Forest plot of meta-analysis: Hospital and surgery outcomes

Compared to HA, THA has longer surgery time (WMD = 20.044, *P* < 0.0001), more blood loss (WMD = 69.106, *P* < 0.0001), and longer hospital length (WMD = 2.360, *P* = 0.031). Fifteen studies reported surgery time (THA 1292, HA 1341) while nine studies reported hospital length (THA 418, HA 443) with high heterogeneity (*I*^2^ = 96%). We further did the Galbraith test and found the main source of the heterogeneity ([25]; H. H [27].). We excluded them, and the results are stable with no heterogeneity (*I*^2^ = 0%). For blood loss, nine studies were included (THA 1063, HA 1038), and the results are stable after removing developing countries’ studies (Fig. 4).

##### Clinical outcomes

**Fig. 5:**
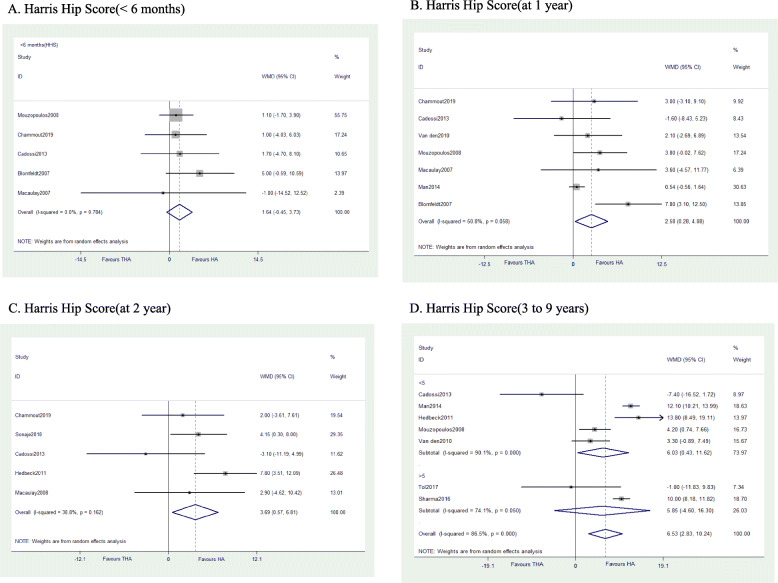
Forest plot of meta-analysis: Harris Hip Score

**Fig. 6:**
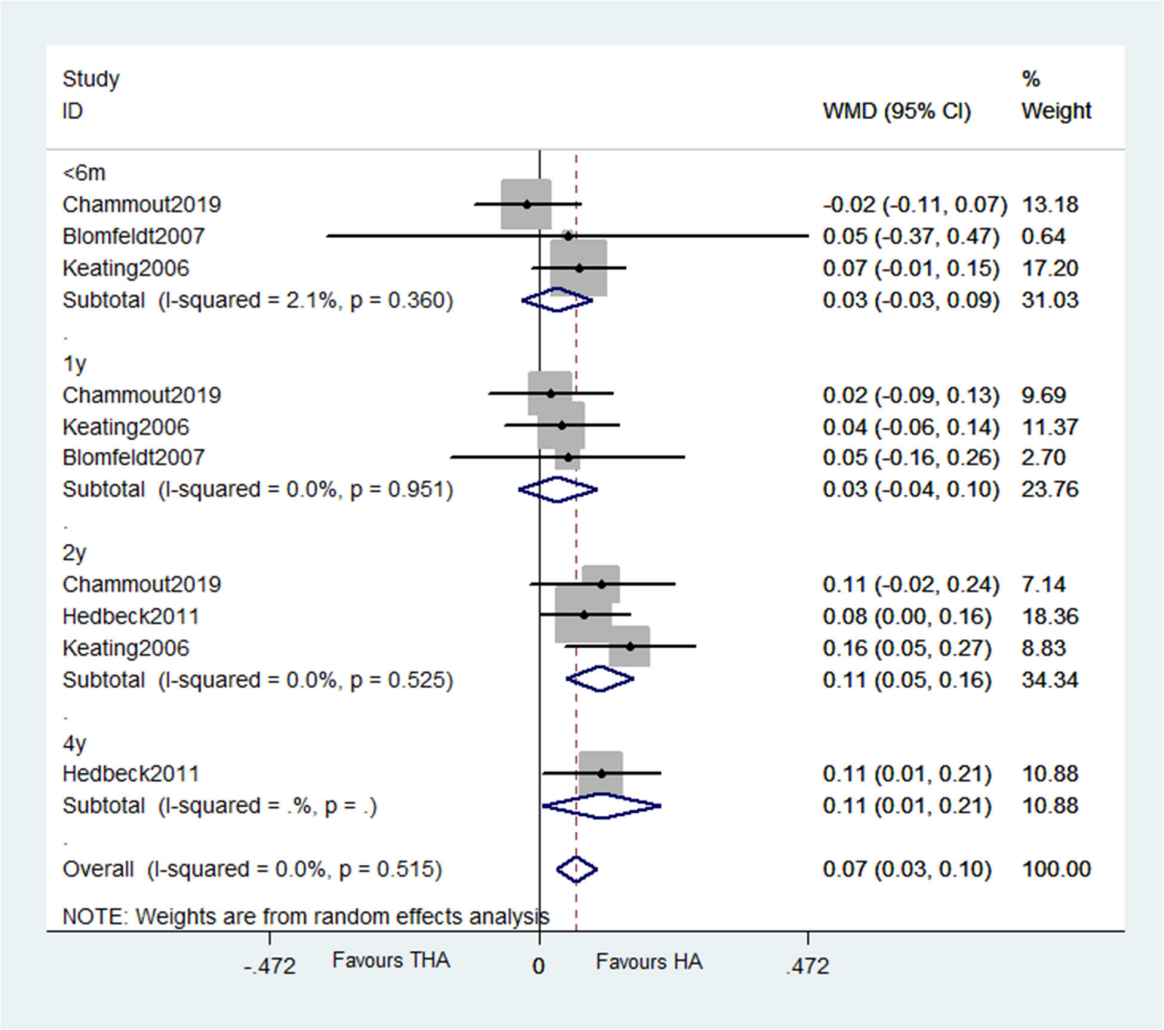
Forest plot of meta-analysis: EQ–5D

The results evidenced THA has similar HHS score with HA within 6 months (WMD = 1.641, *P* = 0.124) or after 9 years (WMD = 5.848, *P* = 0.273) but higher scores at 1 year (WMD = 3.593, *P* = 0.002), 2 years (WMD = 3.691, *P* = 0.020), and 3 to 5 years (WMD = 6.027, *P* = 0.035) (Fig. 5). Three studies reported pain score based on HHS subscore, and other three studies reported pain as binary variables; the results of both show no difference between groups at any follow-up points. For patients’ quality of life, pooled data revealed no significant difference of EQ-5D scores up to 1 year after surgery. But the results favor THA 2 years later (WMD = 0.107, *P* < 0.0001) (Fig. 6).

##### Patients’ quality of life

The results showed that EQ-5D scores within 6 months (WMD = 0.031, *P* = 0.324) and at the first year after surgery (WMD = 0.033, *P* = 0.351) are similar between groups while favor THA 2 years later (WMD = 0.107, < 0.0001).

##### Common complications

The pooling data elicited no statistical difference across groups in terms of the events of pulmonary embolism, deep vein thrombosis, pneumonia, pressure injury, wound disease, surgical-site infection, and cardiovascular disease.

##### Prothesis-related complications

**Fig. 7:**
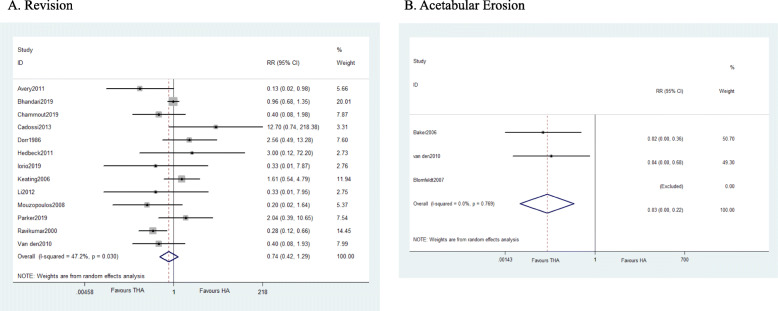
Forest plot of meta-analysis: Prosthesis-related complications

A total of 13 studies suggested that revision rate is similar in both groups with a moderate heterogeneity (*I*^2^ = 47.2%), the Galbraith test detected the main source, and the results are stable after deleting the study [40] (*I*^2^ = 30.3%). The study reported a result of 13 years follow-up thus generate the heterogeneity. Sixteen studies evidenced that THA has higher dislocations rate than HA with significant difference (WMD = 1.897, *P* = 0.002). Compared with THA, HA has a higher rate of acetabular erosion (WMD = 0.030, 95% CI 0.004 to 0.219, *P* = 0.001) (Fig. 7). As for fracture, loosening or subsidence, and heterotopic ossification, the results detected no statistical difference between groups.

##### Mortality

**Fig. 8:**
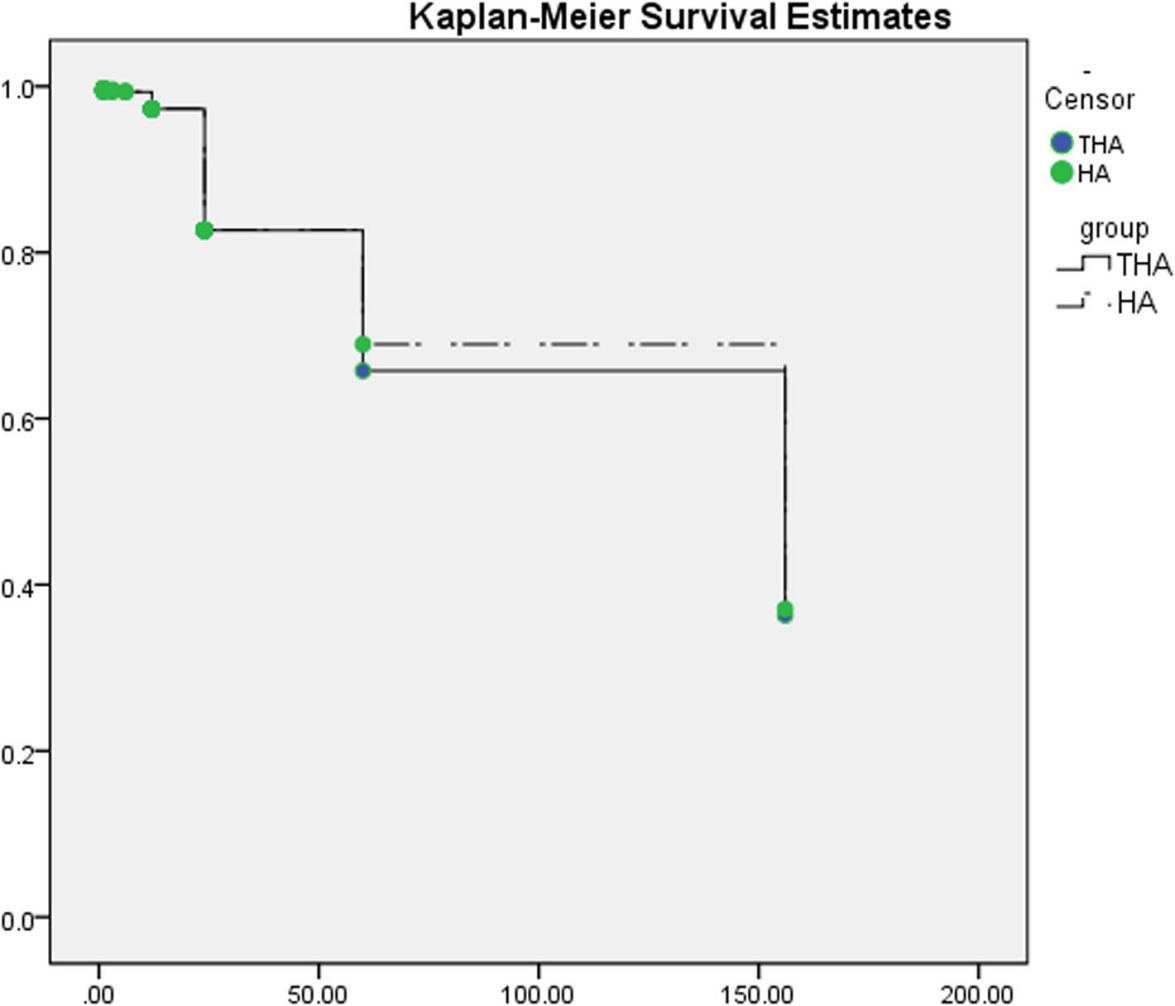
Survival curve

The Kaplan–Meier curve was applied, and we detected the similarity of survivorship (HR 1.029; 95% CI 0.905 to 1.169; *P* = 0.665; Fig. 8). Subgroup analysis of 2 years follow-up revealed reduced mortality in HA group (WMD = 1.224, *P* = 0.008)

## Discussion

### Hospital and surgery outcomes

For surgery time, almost all previous synthesized outcomes are in consistence with our results [24, 28, 33, 48, 49]. And we consider the main reasons are that HA requires less operative installation steps including cup preparation and implantation. For hospital length, we found that THA has longer in-hospital duration in our study. The common reasons for delayed discharges are usually post-surgery complications, since we did not find out the difference in common complications, and we consider that the early ambulation ability for patients who undergone HA may cause the difference. We also found reduced blood loss in HA group and less surgical procedures; tissue damage may clarify the results.

All three indicators are in favor of HA group, and the results are hardly to change even with more evidence. However, the results may lack clinical values when it comes to decision-making.

### Clinical outcomes

Many studies have proved better outcomes in THA group in terms of HHS but did not provide long-term results or subgroup analysis due to limited trials ([24]; Y [28, 33, 49].). We made subgroups based on follow-up periods and initially found that THA group has higher total HHS in medium term (1–5 years) but no difference in short (< 6 months) or long terms (> 9 years).

For pain scores, we detect no difference between two groups, and the PCU-THA used in one trial is the main source of heterogeneity [6]. Liu (Y [28].) and Wang [20] found that patients in THA group experienced significantly less pain, but they only include limited trials in the pooled results.

### Patients’ quality of life

For EQ-5D scores, our conclusion agreed with other studies that THA has better overall patients’ quality of life ([24]; Y [28, 48, 49].). We did the subgroup analysis and found that the difference became obvious 2 years after the surgery.

### Common complications

Our result found no difference in terms of common complications, and we believed further studies can hardly change it. Our results are against Liu et al.’s study (Y [28].). In his study, he limited patients’ age to over 75 years old, and we believe the complications may largely be attributed to the patients’ own condition rather than implants type.

### Prothesis-related complications

The results show that revision rate is similar with moderate heterogeneity (*I*^2^ = 47%). After sensitivity analysis, Ravikumar and Marsh’s [41] study was considered as the source because they reported 13-year follow-up results (24% in HA; 6.75% in THA). In meta-analysis that only include RCTs, Metcalfe et al. [32], Liu et al. (Y [28].), and Migliorini et al. [33] are in favor of our results but Migliorini et al. found a higher revision rate in THA within 5 years while in HA after 5 years. Lewis et al. [24] found that THA was superior to HA, but the non-RCTs in his study may influence the evidence grades.

However, data from registries are in contrast to the results from randomized trials because RCTs always have certain selections of enrolled patients. According to national registry studies, dislocation, infection, and periprosthetic fracture are the main reasons for revision [35, 45]. Anterolateral approach, cemented stem, bipolar head, and 36-mm cups are useful methods to reduce revisions and should be considered by the surgeons for the best outcomes for patients [14, 32, 35, 45]. Dislocations are always a concern by clinical doctors because they are the main reason for revision. We found that THA has a higher rate of dislocation compared with HA. The types of head (bipolar vs. unipolar), cups (dual-mobility vs. single cup), age of patients, pre-injury ambulation status, and surgical approaches may cause influence on the dislocation rate. Our conclusion is in line with other reviews and registry reports ([19, 24]; Y [28, 33].; ). Acetabular erosion is a theoretical indication to perform a revision in a painful HA. The pooled data shows higher acetabular erosion rate in HA group. And we found no dissent from other authors. Osteoarthritis also represents an important pillar for the decision on therapy.

Usually, surgeons are conservative about THA due to the elevated risk of dislocation, with the associated risk of subsequent revisions and the death risk in the end. However, our results found that the revision rate is similar between two groups. The possible reason is that THA has higher dislocations rates while HA has higher acetabular erosion rates and thus equals the revision rate between the two groups. The long-term results favor the THA, and surgeons could choose propriate implants and approaches to reduce dislocation rates.

### Mortality

We found that the mortality rate was similar in groups, and comparable results were found by other meta-analysis [24, 33, 48, 49]. However, we found that THA has a slightly higher mortality rate 2 years after surgery, and it proves the detective ability of our study. We hypothesize that the early revision caused by dislocations will lead to more deaths in THA group while will be offset by acetabular erosion later. But the result should be interpreted carefully with more studies.

### Cost

Three studies mentioned the cost of both techniques. Burgers et al. [5] found that main cost were rehabilitation fares and nursing home care payments in the first year after surgery. Keating et al. [22] found that the cost between groups was not significant, but highlight the high costs of the readmissions in patients who underwent HA. Ravi et al. [39] found that THA reduced health care costs about the index admission 1 year after surgery, relative to HA. Dangelmajer et al. [9] found that patient’s age and medical care payer status were all associated with odds of receiving THA, and patients with private insurance had higher odds of receiving THA. Reducing costs after hip fracture surgery should focus on improving the duration and efficiency of the rehabilitation phase. The economic evidence showed that THA should be more considered because it can cut the cost of readmission and rehabilitation.

## Limitations

There are some limitations also needed to be noticed. First, lack of information (implant types, operative approach, etc.), uncontrollability of confounders (medical resources, surgeon experience, etc.), and other factors might affect the credibility of the pooled data despite that we selected the most reliable types of trials. Secondly, we did not set strict inclusion criteria since they have already been considered in the process of RCTs, and the low heterogeneity of these results also proves it. Thirdly, despite that our results suggested the difference between short-term and long-term results in functional outcomes and patients’ quality of life, the long-term reports are still limited.

Therefore, the multicentered and large population-based designs of future research should be considered, and more long-term follow-up surveys should be focused and reported.

## Conclusion

Based on the results, we thought HA could be recommended for patients who have cognitive impairment, comorbidities, reduced performance status, and low function demands. And THA should be recommended for patients who are active, healthy, with long life expectancy and young biological age, and have higher demands for functions and quality of life.

## Supplementary Information

**Additional file 1: Supplementary file 1.** Search Strategy

## Data Availability

The datasets used during the current study are available from the corresponding author on reasonable request.

## Abbreviations

RCTs: Randomized controlled trials
THA: Total hip arthroplasty
HA: Hemiarthroplasty
FNFs: Femoral neck fractures
CNKI: Chinese National Knowledge Infrastructure
CBM: China Biology Medicine Disc
RRs: Risk ratios
WMDs: Weighted mean differences
CIs: Confidence intervals
PRISMA: Preferred Reporting Items for Systematic Reviews and Meta-Analyses
EQ-5D: EuroQol Five Dimensions Questionnaire
HHS: Harris Hip Scores
