# Supplementary material for: The comparison between total hip arthroplasty and hemiarthroplasty in patients with femoral neck fractures: a systematic review and meta-analysis based on 25 randomized controlled trials
Source: J Orthop Surg Res. 2020 Dec 10;15:596. doi: 10.1186/s13018-020-02122-6 (PMC7730787; doi:10.1186/s13018-020-02122-6)
Supplement: Supplementary file 1 — Additional file 1: Supplementary file 1. Search Strategy [file 13018_2020_2122_MOESM1_ESM.docx]

Key words:

Entry Terms:

Arthroplasty, Replacement, Hip

Arthroplasties, Replacement, Hip

Arthroplasty, Hip Replacement

Hip Prosthesis Implantation

Hip Prosthesis Implantations

Implantation, Hip Prosthesis

Implantations, Hip Prosthesis

Prosthesis Implantation, Hip

Prosthesis Implantations, Hip

Hip Replacement Arthroplasty

Replacement Arthroplasties, Hip

Replacement Arthroplasty, Hip

Arthroplasties, Hip Replacement

Hip Replacement Arthroplasties

Hip Replacement, Total

Replacement, Total Hip

Hip Replacements, Total

Replacements, Total Hip

Total Hip Replacements

Total Hip Replacement

Entry Terms:

Hemiarthroplasty

Hemiarthroplasties

Hemi-Arthroplasty

Hemi Arthroplasty

Hemi-Arthroplasties

Entry Terms:

Femoral Neck Fractures

Femoral Neck Fracture

Femur Neck Fractures

Femur Neck Fracture

"randomized controlled trial"[pt] OR "controlled clinical trial"[pt] OR randomized[tiab] OR placebo[tiab] OR "drug therapy"[sh] OR randomly[tiab] OR trial[tiab] OR groups[tiab]

'randomized controlled trial'/exp OR 'controlled clinical trial'/exp OR randomized:ti,ab OR placebo:ti,ab OR 'drug therapy':lnk OR randomly:ti,ab OR trial:ti,ab OR groups:ti,ab
